# Which Risk Factors Predict Knee Ligament Injuries in Severely Injured Patients?—Results from an International Multicenter Analysis

**DOI:** 10.3390/jcm9051437

**Published:** 2020-05-12

**Authors:** Christian D. Weber, Lucian B. Solomon, Rolf Lefering, Klemens Horst, Philipp Kobbe, Frank Hildebrand, TraumaRegister DGU

**Affiliations:** 1 Department of Orthopaedics and Trauma Surgery, RWTH Aachen University, 52074 Aachen, Germany; khorst@ukaachen.de (K.H.); pkobbe@ukaachen.de (P.K.); fhildebrand@ukaachen.de (F.H.); 2 Orthopaedic and Trauma Service, Royal Adelaide Hospital and Centre for Orthopaedic and Trauma Research, The University of Adelaide, SA 5005, Adelaide, Australia; bogdansolomon@me.com; 3 Institute for Research in Operative Medicine (IFOM), Witten/Herdecke University, 51109 Cologne, Germany; rolf.lefering@uni-wh.de; 4 Committee on Emergency Medicine, Intensive Care and Trauma Management (Sektion NIS) of the German Trauma Society (DGU), 10623 Berlin, Germany; traumaregister@auc-online.de

**Keywords:** risk factors, knee joint injuries, knee dislocation, ligament injuries

## Abstract

*Introduction:* Ligament injuries around the knee joint and knee dislocations are rare but potentially complex injuries associated with high-energy trauma. Concomitant neurovascular injuries further affect their long-term clinical outcomes. In contrast to isolated ligamentous knee injuries, epidemiologic data and knowledge on predicting knee injuries in severely injured patients is still limited. *Methods:* The TraumaRegister DGU^®^ (TR-DGU) was queried (01/2009–12/2016). Inclusion criteria for selection from the database: maximum abbreviated injury severity ≥ 3 points (MAIS 3+). Participating countries: Germany, Austria, and Switzerland. The two main groups included a “control” and a “knee injury” group. The injury severity score (ISS) and new ISS (NISS) were used for injury severity classification, and the abbreviated injury scale (AIS) was used to classify the severity of the knee injury. Logistic regression analysis was performed to evaluate various risk factors for knee injuries. *Results:* The study cohort included 139,462 severely injured trauma patients. We identified 4411 individuals (3.2%) with a ligament injury around the knee joint (“knee injury” group) and 1153 patients with a knee dislocation (0.8%). The risk for associated injuries of the peroneal nerve and popliteal artery were significantly increased in dislocated knees when compared to controls (peroneal nerve from 0.4% to 6.7%, popliteal artery from 0.3% to 6.9%, respectively). Among the predictors for knee injuries were specific mechanisms of injury: e.g., pedestrian struck (Odds ratio [OR] 3.2, 95% confidence interval [CI]: 2.69–3.74 *p* ≤ 0.001), motorcycle (OR 3.0, 95% CI: 2.58–3.48, *p* ≤ 0.001), and motor vehicle accidents (OR 2.2, 95% CI: 1.86–2.51, *p* ≤ 0.001) and associated skeletal injuries, e.g., patella (OR 2.3, 95% CI: 1.99–2.62, *p* ≤ 0.001), tibia (OR 1.9, 95% CI: 1.75–2.05, *p* ≤ 0.001), and femur (OR 1.8, 95% CI: 1.64–1.89, *p* ≤ 0.001), but neither male sex nor general injury severity (ISS). *Conclusion:* Ligament injuries and knee dislocations are associated with high-risk mechanisms and concomitant skeletal injuries of the lower extremity, but are not predicted by general injury severity or sex. Despite comparable ISS, knee injuries prolong the hospital length of stay. Delayed or missed diagnosis of knee injuries can be prevented by comprehensive clinical evaluation after fracture fixation and a high index of suspicion is advised, especially in the presence of the above mentioned risk factors.

## 1. Introduction

High-energy trauma, in particular, can result in multisystem injuries, which may involve both isolated or multi-ligamentous knee injuries [1,2]. The acute traumatic dislocation of the knee is considered the most severe ligament injury of the lower extremity and may be associated with devastating and limb-threatening complications [3,4,5,6]. The incidence for knee dislocations is very low and has been estimated to be 1.2 per million person-years, mainly from high-energy trauma [7]. However, recent publications also reported knee dislocations as a result of low-energy mechanisms, including ground level falls in obese individuals [8,9,10]. The prompt identification, dedicated evaluation, and a comprehensive management of knee injuries have a high impact on long-term functional outcomes [11,12,13,14].

In fact, ligament injuries around the knee and dislocations of the knee joint have been poorly described in the context of multiple trauma [15]. Previous studies have suggested anatomic risk factors for knee injuries, including femoral shaft fractures [16,17], combined femur and tibia fractures [18], patella fractures [19], and acetabular fractures [20]. According to Byun et al. around 30% of concomitant knee injuries after femoral shaft fracture are not detected in time [17] and frequently become symptomatic within the first posttraumatic year [20]. The mean time for diagnosis of associated knee ligament injury in neglected cases after fixation of a femoral shaft fracture was around 10.6 weeks (range, 1–32) [17].

While a meta-analysis suggested improved outcomes for the early reconstruction of complex knee injuries [21], this concept would be jeopardized by a delayed diagnosis of injuries to the knee. Therefore, the identification of risk factors based on anatomical injury patterns or injury mechanisms might be helpful to guide the diagnostic and surgical strategy, to improve the early detection of knee injuries, and to prevent long-term complications.

In this context, this study was designed as a multi-center registry analysis to increase the knowledge about ligament injuries around the knee and knee dislocations in severely injured trauma patients. We attempted to answer the following questions:

1. What is the incidence for knee ligament injuries and knee dislocations?
2. What are the predominant injury mechanisms leading to knee injuries?
3. What are independent predictors for knee injuries in severely injured trauma patients?

## 2. Patients and Methods

### 2.1. TraumaRegister DGU^®^ and Data Acquisition

The TraumaRegister DGU^®^ of the German Trauma Society (Deutsche Gesellschaft für Unfallchirurgie, DGU) was founded in 1993 [22,23]. The aim of this multi-center database is a pseudonymized and standardized documentation of severely injured patients. Data are collected prospectively in four consecutive time phases from the site of the accident until discharge from hospital: (A) Pre-hospital phase, (B) emergency room and initial surgery, (C) intensive care unit (ICU), and (D) discharge.

The documentation includes detailed information on demographics, injury pattern, comorbidities, pre- and in-hospital management, a course in ICU, relevant laboratory findings including data on transfusion, and the outcome of each individual. The inclusion criterion is admission via the emergency room with subsequent intensive or intermediate care or death before admission to ICU. The infrastructure for documentation, data management, and data analysis are provided by the Academy for Trauma Surgery (AUC—Akademie der Unfallchirurgie GmbH), a company affiliated with the German Trauma Society. The scientific leadership is provided by the Committee on Emergency Medicine, Intensive Care and Trauma Management (Sektion NIS) of the German Trauma Society. The participating hospitals submit their data pseudonymized into a central database via a web-based application. Scientific data analysis is approved according to a peer review procedure established by Sektion NIS. The participating hospitals are primarily located in Germany (90%), but a rising number of hospitals of other countries contribute data as well (at the moment Austria, Switzerland, Belgium, Finland, Luxemburg, Slovenia, The Netherlands, and the United Arab Emirates). Currently, approx. 35,000 cases from almost 700 hospitals are entered into the database per year. Participation in TraumaRegister DGU^®^ is voluntary. For hospitals associated with the TraumaNetzwerk DGU^®^, however, the entry of at least a basic dataset is obligatory for reasons of quality assurance. The present study is in line with the publication guidelines of the TraumaRegister DGU^®^ and registered as TR-DGU project ID 2014-057.

### 2.2. Inclusion and Exclusion Criteria

This study included data from all patients included in the TraumaRegister DGU^®^ with severe injuries (MAIS 3+) after admission to a participating trauma center in Germany, Austria, or Switzerland between January 2009 and December 2016.

Patients transferred *out* to another center within 48 h after admission were excluded because of missing outcome data and to exclude the risk of a double counting from the receiving hospital. However, all cases transferred *in* were included to prevent bias in prevalence rates.

### 2.3. Definitions

#### 2.3.1. Mechanism of Injury

According to the TR-DGU dataset, the following injury mechanisms were considered: (1) Motor vehicle accident (MVA), (2) motorcycle accident (MCA), (3) bicycle accident, (4) pedestrian struck by vehicle, (5) high fall (≥ 3 m), and (6) low fall (< 3 m); further (combined) categories include (7) suicide attempt, (8) other (not shown), (9) blunt/penetrating trauma (not shown), and (10) traffic-related (overall value presented).

#### 2.3.2. Injury Severity

Since 2009, coding follows a uniform protocol, and the data management has been previously described [23]. All injuries were coded according to the Abbreviated Injury Scale (AIS Version 2005/Update 2008, Association for the Advancement of Automotive Medicine, Barrington, IL) [24,25]. According to the AIS, the severity of injuries was documented as follows [26]: 1 (minor), 2 (moderate), 3 (severe, not life-threatening), 4 (serious, life-threatening), 5 (critical, survival uncertain), or 6 (maximum, currently untreatable). The injury severity score (ISS) and the new ISS (NISS) were derived from documented AIS values [26,27].

#### 2.3.3. Identification of Knee Injuries and Group Distribution

Identification of ligament injuries according to AIS codes: (1) AIS 8740**.1: knee ligament injury (including subluxation); (2) AIS 87403*.2: (multi-)ligament injury with knee dislocation; and (3) AIS 84080*.2: partial or complete ligament rupture. Neurovascular injuries around the knee were identified independently from ligamentous injuries either as a popliteal artery injury (AIS 8206**.2) and/or a peroneal nerve injury (AIS 8305**.2).

Patients with one of the aforementioned AIS codes were assigned to the “knee injury” group, whereas patients without these injuries were included in the “control” group.

## 3. Statistical Analysis

Categorical data were presented as frequencies and percentages. Metric variables were reported as means and standard deviations (SD). For skew distributed data, the median is also reported. Severe trauma patients without a ligament injury of the knee served as a control group. Formal statistical testing was avoided because of the very large sample size. Multivariate logistic regression analysis was performed to evaluate the impact of various risk factors for knee injuries. Results are presented as odds ratios (OR) with 95% confidence intervals. The analysis was performed with SPSS (Version 25, IBM Inc., Armonk, NY, USA).

## 4. Results

### 4.1. Demographic Data and Mechanism of Injury

During the study period, 139,462 severely injured patients fulfilled the inclusion criteria. Of these, 4411 patients sustained a knee injury (3.2% overall incidence), and 1152 patients (0.8%) suffered from a multi-ligament injury related to a knee dislocation. Ligamentous knee injuries in polytraumatized patients were most commonly caused by road traffic accidents (84.8%, Table 1). MVA and MCA were equally responsible for 62% of these (1375 patients or 31.7% for MVA and 1305 or 30.1% for MCA), while pedestrians struck by vehicles were responsible for 13.3% of these injuries (578 patients).

**Figure 1:**
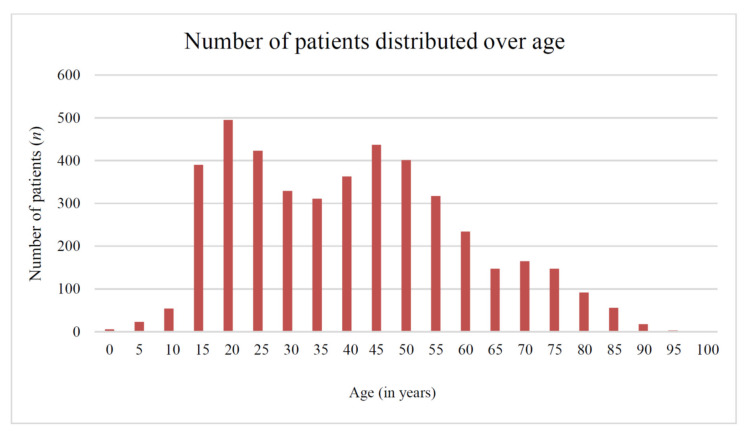
Age distribution of patients with knee ligament injuries (each column represents a 5-year interval).

Of the patients, 98,252 (70.7%) were male (Table 2). The “knee injury” group had a mean age of 43 years (±19), and the control group had a mean age of 52 years (±22). The vast majority of patients with knee ligament injuries were between 16 and 59 years of age (n = 3447, 78.1%). Pediatric and elderly patients infrequently sustained ligamentous knee injuries. The age distribution was found bimodal; we observed two peaks: 20–30 years and 40–50 years (Figure 1).

### 4.2. Injury Pattern

Mean ISS (21.7 vs. 21.4 points) and NISS (27.5 vs. 26.0 points) values were comparable for both groups. In terms of associated injuries, the control group had a higher rate of head injuries (45.0%) compared to the knee injury group (25.9%).

No clinically relevant variance for facial, chest, and abdominal or spinal injuries was observed (Table 3) between the two groups. However, upper and lower extremity injuries and pelvic fractures were more frequently diagnosed in patients with ligamentous knee injuries. The rate of associated neurovascular injuries was increased in patients with ligamentous knee injuries and even more pronounced in case of knee dislocation (peroneal nerve: 6.7% and popliteal artery: 6.9%).

### 4.3. Length of Hospital Stay and Delayed Diagnosis

The hospital stay was prolonged by seven days in patients with associated ligamentous knee injuries, and 228 patients had a delay longer than 48 h in the diagnosis of a ligamentous knee injury.

### 4.4. Multivariate Logistic Regression Analysis

The multivariate logistic regression analysis model (Table 4) identified various variables associated with an increased risk for knee ligament injury: (1) Pedestrian struck by vehicle (OR 3.2, 95% CI: 2.69–3.74 *p* ≤ 0.001); (2) MCA (OR 3.0, 95% CI: 2.58–3.48, *p* ≤ 0.001); (3) patella fracture (OR 2.3, 95% CI: 1.99–2.62, *p* ≤ 0.001); (4) MVA (OR 2.2, 95% CI: 1.86–2.51, *p* ≤ 0.001); (5) tibia fracture (OR 1.9, 95% CI: 1.75–2.05, *p* ≤ 0.001); (6) femur fracture (OR 1.8, 95% CI: 1.64–1.89, *p* ≤ 0.001); (7) fibula fracture (OR 1.8, 95% CI: 1.62–1.98, *p* ≤ 0.001); (8) bicycle accident (OR 1.5, 95% CI: 1.21–1.74, *p*≤ 0.001); (9) pelvic fracture (OR 1.3, 95% CI: 1.24–1.44, *p* ≤ 0.001); and (10) upper extremity fracture (OR 1.1, 95% CI: 1.03–1.18, *p* = 0.004).

## 5. Discussion

This study interrogated a large registry database to investigate the mechanisms of injury that cause associated ligamentous knee injuries in severely injured patients. Furthermore, demographics and associated injuries in severely injured patients with knee ligament injuries were characterized. Such information is likely to support the development of diagnostic protocols and thereby to increase the early diagnosis of ligamentous knee injuries in this patient cohort. This is of upmost importance because it is known that such injuries are missed in up to 30% of patients [17], and a delayed diagnosis of these injuries can have devastating effects on their outcomes [28]. In a long-term study after multiple trauma, knee injuries were most often responsible for preventing individuals to return to sports [13].

### 5.1. Incidence and Demographic Data

The incidence of ligamentous knee injuries in our study population was 3.2%, and 0.8% of the included patients suffered a dislocated knee. These findings are in line with recent publications, since ligamentous knee injuries, especially knee dislocations, are rare injuries. Particularly, traumatic knee dislocations are very rare and have been described to account for only 0.02% of orthopedic injuries [29].

We observed a bimodal age distribution for ligamentous knee injuries, with one peak in the third decade of life and a second peak in the fifth decade of life. The highest incidence of ligamentous knee injuries in the severely injured patient was previously identified in men aged between 18 and 29 years (incidence, 29 per 1 million person-years in 2011) [30]. The observed bimodal distribution with a second peak in the fifth life decade may be caused by the current demographic situation in the participating countries [31,32], and therefore might also reflect the reality of social and behavioral changes in people’s lives at certain time points.

### 5.2. Mechanisms and Patterns of Injury

Recently, a number of publications highlighted low-velocity mechanisms in obese individuals as novel and separate mechanism causing ligamentous knee injuries [7,8,9,10]. According to our data, low energy falls contributed to only 4.9% of all ligamentous knee injuries, and the vast majority of complex knee injuries were associated with high-energy trauma. Predominant injury mechanisms leading to knee injuries were MVAs, MCAs, and pedestrian struck by a car. Multiple previous studies also reported motor vehicle collisions as the major cause of these injuries [33,34,35,36].

### 5.3. Associated Injuries and Outcome

While we report ligamentous and neurovascular injuries of the knee in dichotomous fashion, we are unable to comment on exact intra-articular pathologies. Krych et al. evaluated 122 dislocated knees in 121 patients and found associated intra-articular injuries in addition to ligament injuries in 76% of patients [37].

We identified a high incidence of concomitant injuries particularly of the lower extremity (i.e., patella fractures) and the chest in our population with ligamentous knee injuries. This finding should prompt a comprehensive assessment of ligamentous knee injuries in all these situations, especially when the knee injury is not obvious. Accordingly, Darcy et al. [29] reported in a group of 80 patients with knee dislocation that 57.1% of the cases were associated with another injury. Also other reports described a number of injuries associated with ligamentous knee injuries. In this context it was found, that 25% of patellar fractures were associated with a posterior cruciate ligament injury [19]. A retrospective analysis of 114 femoral shaft fractures in 110 patients, at an average of 3.9 years post-injury, showed that 27% of patients had some form of knee ligament insufficiency, while 11% complained of knee instability [18]. The same study found that knee instability was even more prevalent, with 54% in cases of combined ipsilateral femur and tibial shaft fractures [18]. As a conclusion, the authors advocated examination of the knee under anesthesia in all patients with a femoral shaft fracture. Accordingly, another study found a 20% risk of ipsilateral ligamentous knee injuries after femoral shaft fractures (87 of 429 cases, 32 of which were multi ligamentous injuries) [17]. Most importantly, 30% of these injuries were only detected at an average of 11 weeks (range 1–32 weeks) after the surgical treatment of the femoral shaft fracture. At 14.7%, the delayed diagnosis of ligamentous knee injuries in our study was half of that.

### 5.4. Associated Neurovascular Injuries

This study found that the incidence of popliteal artery injuries associated with ligamentous injuries to the knee is bigger than previously reported by large cohort series. Notably, popliteal artery injuries can also be associated with isolated ligamentous injuries of the knee, as our study identified 99 popliteal artery injuries (2.2%) in patients with isolated knee ligamentous injuries. The incidence of popliteal artery injury in patients with knee dislocations at 6.9% (79 cases) in our study was higher than in other studies with large cohorts based on either an analysis of a large insurance database [38] or a prospective review of 303 cases of knee dislocations [39]. This might be explained by a higher proportion of high energy trauma cases in our study. However, the incidences of our and the other aforementioned studies are markedly lower compared to results from smaller cohort series investigating knee dislocations. For example, a review of 67 patients at a multicenter study found the incidence of popliteal artery injury in 12% (9 patients) of patients with knee dislocations in which both cruciate ligaments are injured [40], while a systematic review of 23 studies with rather small study populations with knee dislocation found the incidence of popliteal artery injury to be 18% [3]. Excluding the questions related to relatively small retrospective studies and the associated selection bias, it is possible that such differences are also related to the mechanism of injury and patient-specific factors. In this context, the exact role of the injury mechanism for the development of popliteal injuries is not fully clarified. While this and other studies [3,10,30,41,42,43] mainly observed vascular injuries associated with knee dislocations in high-energy trauma cases, some authors suggest that knee dislocation-associated vascular injuries preferably appear secondary to low energy injuries (21% in ultra-low energy injuries) when compared with high energy injuries mechanisms (13%) [41]. Also patient- and injury-specific risk factors (increased body mass index and open injuries, OR 3.366; 95% CI, 1.008–11.420; *p* = 0.048) for vascular injuries in case of knee dislocation were identified [42]. This study also found that the incidence of peroneal nerve injuries was smaller than previously reported. We identified 148 (3.9%) peroneal nerve injuries in patients with isolated knee ligamentous injuries and 77 peroneal nerve injuries (6.7%) in patients with knee dislocations. By comparison, a prospective review of 303 patients with knee dislocations found the incidence of peroneal nerve injuries to be 19.2% [39], and a systematic review identified the risk of common peroneal nerve injuries in knee dislocations to be as high as 25% [3]. As with the vascular injuries, injury mechanism and patient-specific factors might again explain these differences. In this context, some literature has suggested that lower energy injuries are more likely to be associated with nerve damage compared with higher energy injuries [41].

### 5.5. Limitations

This study has several limitations. Owing to the nature of the TraumaRegister DGU^®^ and the well-known inclusion criteria, our data concerning injury mechanisms were biased toward high-energy injuries rather than low-energy injuries. Therefore, our findings are not generalizable to isolated injuries such as ultra-low-energy knee dislocations, e.g., from low-level falls. Furthermore, as the number and anatomic sites of ligament injuries around the knee are not consistently documented within the TraumaRegister DGU^®^, we are unable to report the exact anatomic injury patterns. Additionally, in severely injured patients, there is a risk of underreporting of knee injuries related to early mortality or limb-loss. The TraumaRegister DGU^®^ is also not designed to capture occult knee injuries that were diagnosed after hospital discharge or transfer to a rehabilitation facility. Especially in patients with limited mobility, knee instability, and associated pain might be clinically unapparent prior to weight-bearing mobilization during rehabilitation. However, the current study also has several strengths to be recognized. First, to our knowledge, this is the largest study analyzing knee injuries in severely injured patients. Therefore, the epidemiologic data, description of injury mechanisms, general injury severity, and concomitant injuries seem to be valid and clinically relevant. Second, the logistic regression analysis involved a wide range of risk factors including injury mechanisms and skeletal injuries. The analysis identified various independent predictors for knee injuries that are also applicable in sedated and ventilated trauma patients.

## 6. Conclusions

Ligamentous knee injuries caused by high-energy trauma are associated with concomitant injuries of the pelvis, femur, patella, and lower leg, but are not predicted by general injury severity or sex. Despite comparable injury severity, ligamentous knee injuries prolong the hospital length of stay and could therefore be considered a surrogate for increased treatment complexity and costs.

## Figures and Tables

**Table 1:** Mechanisms of injury.

	Control Group *n* = 131,483	Knee Injury *n* = 4340	Total
Motor vehicle collision % (*n*)	20.6% (27,054)	31.7% (1374)	20.9% (28,428)
Motorcycle accident % (*n*)	12.5% (16,391)	30.1% (1305)	13% (17,696)
Bicycle accident % (*n*)	8.5% (11,177)	6.1% (263)	8.4% (11,440)
Pedestrian struck % (*n*)	6.6% (8618)	13.3% (578)	6.8% (9196)
High Fall ≥3~m % (*n*)	17.6% (21,938)	8.5% (367)	16.4% (22,305)
Low Fall <3~m % (*n*)	24.1% (31,667)	4.9% (212)	23.5% (31,879)
Traffic-related (all) % (*n*)	51.7% (64,859)	84.8% (3580)	52.7% (68,439)
Suicide attempt (any) % (*n*)	4.8% (6221)	3.0% (128)	4.7% (6349)

**Table 2:** Demographic and outcome data.

	Control Group	Knee Injury
Male sex % (*n*)	70.5% (94,981)	74.3% (3271)
Mean age (SD), years	52 (22)	43 (19)
Pediatric age (≤ 15 years)	3.3% (4420)	2.3% (102)
ISS: mean (SD), points	21.7 (12)	21.4 (11)
New ISS: mean (SD), points	27.5 (14)	26.0 (12)
ICU LOS: days †	7.4 / 3 (11)	8.4 / 3 (12)
Hospital LOS: days †	19 / 14 (19)	26 / 21 (22)
Delayed injury identification β	8.3% (108)	14.7% (120)

LOS = length of stay; ICU = intensive care unit. (†) mean / median (SD). (β) Injury diagnosed at ICU or later; available for cases with standard documentation only.

**Table 3:** Patterns of injury.

	Control Group	Knee Injury	*Total*
Head injury (AIS ≥3)	45.0% (60,755)	25.9% (1,141)	44.4% (61,896)
Facial injury (AIS ≥2)	11.6% (15,634)	10.9% (483)	11.6% (16,117)
Chest injury (AIS ≥3)	47.7% (64,458)	50.7% (2,238)	47.8% (66,696)
Abdominal injury (AIS ≥3)	12.4% (16,800)	12.1% (532)	12.4% (17,332)
Extremity injury (AIS ≥3)	30.1% (40,674)	56.9% (2,508)	31.0% (43,182)
Spinal injury (AIS ≥2)	29.6% (39,926)	29.1% (1,283)	29.5% (41,208)
UE injury (AIS ≥2)	30.0% (40,563)	39.9% (1,758)	30.3% (42,321)
LE injury (AIS ≥2)	26.3% (35,542)	76.5% (3,375)	27.9% (38,917)
Pelvic injury (AIS ≥2)	17.9% (24,139)	27.1% (1,195)	18.2% (25,334)
Femur fracture % (*n*)	13.9% (18,826)	31.6% (1,394)	14.5% (20,220)
Tibia fracture % (*n*)	10.3% (13,853)	31.7% (1,399)	10.9% (15,252)
Patella fracture % (*n*)	4.5% (6,084)	16.6% (731)	4.9% (6,815)
Peroneal nerve injury % (*n*)	0.4% (489)	3.9% (63)	0.4% (552)
Popliteal artery injury % (*n*)	0.2% (228)	2.2% (99)	0.2% (327)

UE = upper extremity; LE = lower extremity.

**Table 4:** Logistic regression analysis: predictors for ligamentous knee injuries.

Risk Factors	Odds Ratio (OR)	95% Confidence Interval (CI)	*p*-Value
Mechanisms of Injury			
Motor vehicle accident	2.2	1.86–2.51	≤ 0.001
Motorcycle accident	3.0	2.58–3.48	≤ 0.001
Pedestrian struck	3.2	2.69–3.74	≤ 0.001
Bicycle accident	1.5	1.21–1.74	≤ 0.001
Skeletal Injuries			
Pelvic fracture	1.3	1.24–1.44	≤ 0.001
Femur fracture	1.8	1.64–1.89	≤ 0.001
Patella fracture	2.3	1.99–2.62	≤ 0.001
Tibia fracture	1.9	1.75–2.05	≤ 0.001
Fibula fracture	1.8	1.62–1.98	≤ 0.001
Upper extremity fracture	1.1	1.03–1.18	0.004
Demographic Data			
Male sex	1.0	0.96–1.12	0.29
